# Cryo-electron microscopy of extracellular vesicles from cerebrospinal fluid

**DOI:** 10.1371/journal.pone.0227949

**Published:** 2020-01-30

**Authors:** Anton Emelyanov, Tatiana Shtam, Roman Kamyshinsky, Luiza Garaeva, Nikolai Verlov, Irina Miliukhina, Anastasia Kudrevatykh, Gaspar Gavrilov, Yulia Zabrodskaya, Sofya Pchelina, Andrey Konevega

**Affiliations:** 1 Petersburg Nuclear Physics Institute named by B.P. Konstantinov of National Research Center «Kurchatov Institute», Gatchina, Russia; 2 Pavlov First Saint Petersburg State Medical University, St. Petersburg, Russia; 3 National Research Center “Kurchatov Institute”, Moscow, Russia; 4 Shubnikov Institute of Crystallography of Federal Scientific Research Centre “Crystallography and Photonics” of Russian Academy of Sciences, Moscow, Russia; 5 Moscow Institute of Physics and Technology, Dolgoprudny, Moscow region, Russia; 6 Peter the Great St. Petersburg Polytechnic University, St. Petersburg, Russia; 7 Institute of Experimental Medicine, St. Petersburg, Russia; 8 S.M. Kirov Saint-Petersburg Military Medical Academy, St. Petersburg, Russia; 9 Polenov Neurosurgical Institute–Branch of National Almazov Medical Research Centre, St. Petersburg, Russia; Universita degli Studi di Torino, ITALY

## Abstract

Extracellular vesicles (EVs) are membrane-enclosed vesicles which play important role for cell communication and physiology. EVs are found in many human biological fluids, including blood, breast milk, urine, cerebrospinal fluid (CSF), ejaculate, saliva etc. These nano-sized vesicles contain proteins, mRNAs, microRNAs, non-coding RNAs and lipids that are derived from producing cells. EVs deliver complex sets of biological information to recipient cells thereby modulating their behaviors by their molecular cargo. In this way EVs are involved in the pathological development and progression of many human disorders, including neurodegenerative diseases. In this study EVs purified by ultracentrifugation from CSF of patients with Parkinson’s disease (PD) and individuals of the comparison group were characterized using nanoparticle tracking analysis, flow cytometry and cryo-electron microscopy. Vesicular size and the presence of exosomal marker CD9 on the surface provided evidence that most of the EVs were exosome-like vesicles. Cryo-electron microscopy allowed us to visualize a large spectrum of extracellular vesicles of various size and morphology with lipid bilayers and vesicular internal structures. Thus, we described the diversity and new characteristics of the vesicles from CSF suggesting that subpopulations of EVs with different and specific functions may exist.

## Introduction

Extracellular vesicles (EVs) are membrane-enclosed vesicles released by cells in response to various stimuli. EVs are found in many human biological fluids, including plasma, breast milk, urine, saliva, cerebrospinal fluid (CSF), etc. [1]. There are two main types of cell-derived vesicles: microparticles originated from cell plasma membranes (from 100 nm to 1μm), and exosomes that originate from late endosomes (from 40 to 150 nm) [2] secreted by the fusion of multivesicular bodies (MVB) with the plasma membrane. Here, the term EVs is used for designating all types of sub-cellular particles in CSF that are surrounded by a lipid membrane bilayer. EVs are known to carry many different proteins and large amount of nucleic acids, including mRNAs, microRNAs (miRNAs), long non-coding RNAs (lncRNAs) and DNA [2, 3] that may represent the functions of their original cells and could be involved in the presentation of antigens, cell to cell communication including protein propagation [2, 4, 5]. To date, some role of EVs both in normal physiology and in disease pathology was shown [4, 5]. EVs could be involved in the pathological development and progression of numerous diseases including neurodegenerative diseases and in particular Parkinson's disease (PD) [5, 6, 7]. PD is the second most frequent neurodegenerative disorder after Alzheimer’s disease associated with alpha-synuclein aggregation in dopaminergic neurons in the substantia nigra [8, 9]. Some studies have shown that alpha-synuclein aggregates are transmissive and able to spread from cell to cell via exosomes [2, 7]. On the other hand, it is assumed that exosomes could carry other factors that initiate alpha-synuclein oligomerization in recipient cells. In support of this, the lipid composition of exosomes was shown to affect alpha-synuclein aggregation [10]. Interestingly, biological signal transmission may depend on size and morphology of EVs, affecting convective and diffusive transport mechanisms [11]. Definition of size and morphology is important for analyzing EV participation in the intercellular signaling pathways in pathology and normal state. Detailed characterization of CSF vesicles seems to be the most relevant for understanding of their role in the pathogenesis of neurodegenerative disorders.

Currently, a powerful tool for assessing morphology of EVs is cryo-electron microscopy (cryo-EM), which preserves membranes in a close to native state [12]. Detailed characterization of EVs from different body fluids such as blood plasma, breast milk and ejaculate has been conducted via cryo-electron microscopy in recent years [6, 13–17]. However, EVs extracted from CSF remained poorly characterized. In this study we assessed the morphology and size of EVs from CSF using cryo-EM.

## Materials and methods

### Participants

CSF specimens from seven PD patients (age 67.6±7.8, 2 women and 5 men) were collected at the Federal State Budgetary Scientific Institution «Institute of Experimental Medicine» by lumbar tap-test. This procedure was performed for differential diagnosis and exclusion of comorbid pathology (normal pressure hydrocephalus) in PD patients with suspected violation of liquor dynamics. The comparison group consisted of 7 patients not suffering from parkinsonism (age 48±7 years, 3 women and 4 men) and included patients with neurosurgical pathology: 1 epidermoid cyst, 2 vasoneural conflict, 2 posthypoxic encephalopathy, 1 subarachnoid hemorrhage, 1 arachnoid cyst. Cerebrospinal fluid sampling was performed according to the indications associated with the underlying disease.

Specimens were collected in polypropylene tubes and centrifuged at 2,000 g for 10 min at room temperature, aliquoted and frozen at -80 ^0^C until analysis. All experiments were approved by the Ethics Committees of the Institute of Experimental Medicine (Saint-Petersburg, Russia) and Polenov neurosurgical institute–branch of National Almazov Medical Research Centre (Saint-Petersburg, Russia). Signed informed consent was obtained from all PD patients and individuals of comparison group.

### Isolation of EVs

EVs were isolated from CSF samples (5 mL diluted with phosphate-buffered saline (PBS)) using the method described earlier [18]. After preliminary removal of cellular debris and large vesicles by centrifugation (2,000 g for 30 min, and then 16,000 g for 30 min), ultracentrifugation (Beckman Coulter centrifuge, Ti45 rotor) at 110,000 g for 2 h was performed. After centrifugation, the supernatant was removed and the pellet was re-suspended in 0.5 mL of PBS for at least 1 h at 4°C. Then 50 μL aliquots of the resuspended particles were taken for nanoparticle tracking analysis (NTA) and flow cytometry analysis. To prepare the sample for cryo-EM, the remaining volumes of all suspensions of CSF vesicles from either PD group or the comparison group were pooled into two samples and re-centrifuged at 110,000 *g* for 2 h (SW 55Ti rotor). The resulting pellets (preparation of EVs) were dissolved in 40 μL of PBS, rapidly frozen in liquid nitrogen, aliquoted and stored at -80°C until analysis.

### NTA

The size and concentration of EVs were determined by NTA using the NTA NanoSight® LM10 (Malvern Instruments) analyzer, equipped with a blue laser (45 mW at 488 nm) and a C11440-5B camera (Hamamatsu Photonics K.K., Japan). Recording and data analysis were performed using the NTA software 2.3. The following parameters were evaluated during analysis of recording monitored for 60 s: the average hydrodynamic diameter, the mode of distribution, the standard deviation, and the concentration of vesicles in the suspension. Before NTA measuring, an aliquot of the isolated vesicles was thawed at room temperature and diluted with deionized water in 10, 100, 1000 times. The measurements were performed at least three times.

### Flow cytometry

The presence of exosomal marker (tetraspanin CD9) on the surface of the isolated EVs was determined by ready to use kit which allows Fluorescence-Activated Cell Sorting (FACS) analysis of exosome markers (Lonza, Estonia). An aliquot (10 μL) of the isolated EVs was thawed at room temperature. Exosome binding on FACS-beads was performed by incubation at 4°C over night. Exosome-bead complex was labeled with primary antibodies against CD9 and secondary Alexa488-conugated antibodies according to the manufacturer recommendations. A sample without any EVs was used as a negative control for non-specific labeling. An aliquot (10 μL) of Exosome Standard from plasma entering the kit was used as a positive control. Analysis was performed with CytoFlex instrument (Beckman Coulter, USA).

### Cryo-EM

Cryo-EM was used for direct visualization of vesicles. To prepare samples for cryo-EM study lacey carbon EM grids were glow-discharged (30 s, 25 mA) in Pelco EasiGlow system. An aliquot (3 μL) of the aqueous solution of the sample was applied on to the carbon side of EM grid, which was then blotted for 2.0 s and plunge-frozen into the precooled liquid ethane with Vitrobot Mark IV (FEI, USA). This procedure results in embedding the samples in a thin layer of amorphous ice to preserve them in native state and to protect from radiation damages.

The samples were studied in a cryo-electron microscope Titan Krios 60–300 TEM/STEM (FEI, USA), equipped with high sensitive TEM direct electron detector (DED) Falcon II (FEI, USA) and Cs image corrector (CEOS, Germany) at accelerating voltage of 300 kV. To minimize radiation damage during image acquisition low-dose mode in EPU software (FEI, USA) was used. Images were obtained with Falcon II DED at 18000x and 37000x magnification with the defocus value in the range of [-2 μm; -5 μm]. The accumulated total dose per image did not exceed ~50e− /Å^2^.

### Statistical analysis

Conformity of findings to normal distribution was tested using the Shapiro–Wilk test. To assess differences between groups, the Mann–Whitney test was used. The level of significance was set at p < 0.05. Statistical analysis was performed using SPSS 21.0 software. Clinical and experimental data including particle size are expressed as the mean ± the standard deviation (SD) and the median (min-max).

## Results

### Characterization of EVs by NTA and flow cytometry analysis

**Fig 1:**
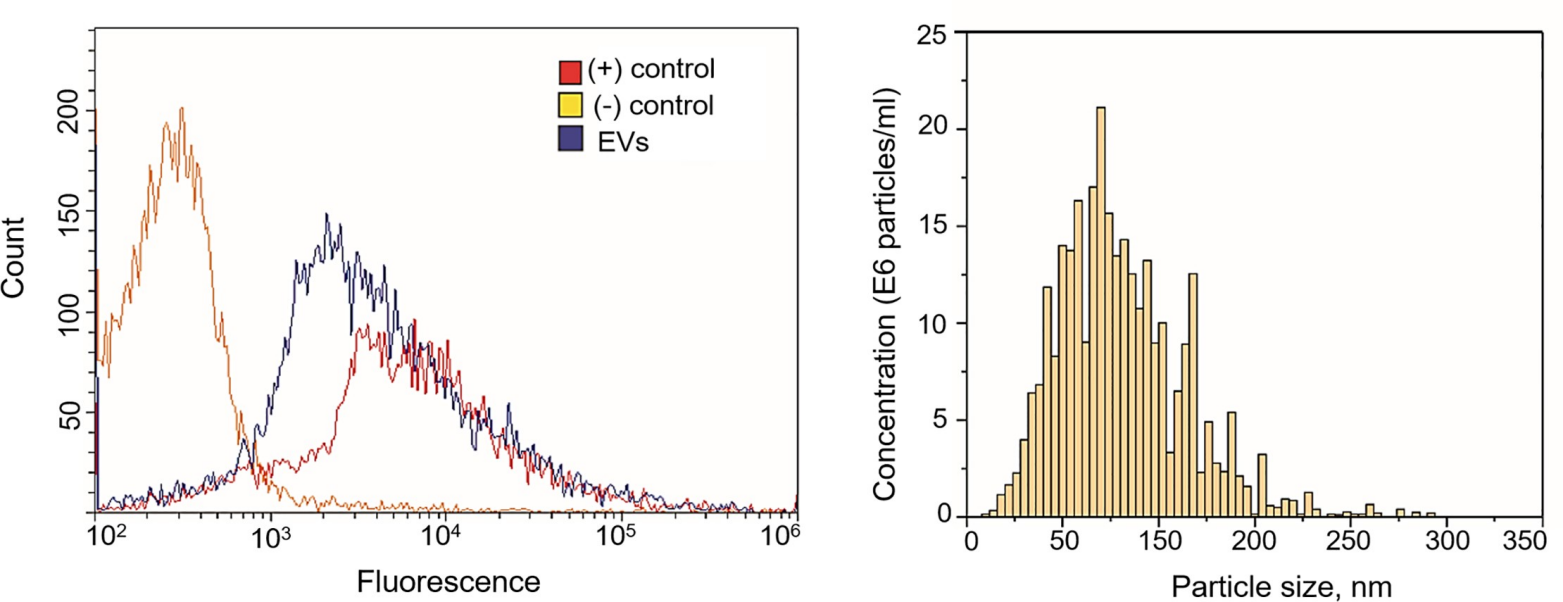
Characterization of EVs isolated from cerebrospinal fluid. (A) Example of Flow cytometric analysis of the CD9 exosomal marker expression on the surface of EVs isolated from CSF (EVs). Immunobeads, which were not incubated with EVs during sample preparation, were used as a negative control ((–) control). The exosomal standard included in the HansaBioMed exosome cytometric assay kit was used as a positive control ((+) control); (B) Nanoparticle tracking analysis (NTA) of particle size and concentration in the sample of vesicles isolated from pooled CSF of PD patients.

EVs were isolated from CSF using ultracentrifugation according the exosome purification protocol described earlier [18]. Characterization of EVs was performed in accordance with International Society for Extracellular Vesicles guidelines [19]. NTA was conducted for EVs derived from CSF of seven PD patients and seven individuals of comparison group. In the samples CSF from PD patients the measured parameters D90 (the diameter at which 90% of the sample's mass is comprised of particles with a diameter less than this value), size mode and concentration of EVs were 217±81 nm, 68±18 nm and (6±5)x10^9^ particles/mL, respectively. In CSF from individuals of the comparison group parameter D90, size mode and concentration of EVs were 214±9 nm, 85±10 nm and (7±1)x10^9^ particles/mL, respectively. No differences in the measured parameters between PD patients and individuals of the comparison group were found. The presence of exosomal marker tetraspanin CD9 was confirmed by flow cytometry in all samples (Fig 1A).

Taking into account the obtained data on the concentration of vesicles in CSF, as well as our own experience in visualization of EVs from different biofluids by cryo-EM [20–22] we pooled all particle samples (from about 30 mL of CSF for PD patients and 50 mL for the comparison group) for re-ultracentrifugation to maximize the number of EVs in the final pellet. NTA of the sample of vesicles isolated from pooled CSF of PD patients showed that the particles generally varied in size from 26 to 305 nm and that the vesicular size mode, D90 and concentration were 69 nm, 125 nm and 0.6x10^12^ particles/ml, respectively (Fig 1B). For CSF pooled from individuals of the comparison group the particle size varied from 22 to 450 nm and the vesicular size mode, D90 and concentration were 77 nm, 244 nm and 2.1x10^12^ particles/ml, respectively.

### Morphological characterization of EVs by Cryo-EM

**Fig 2:**
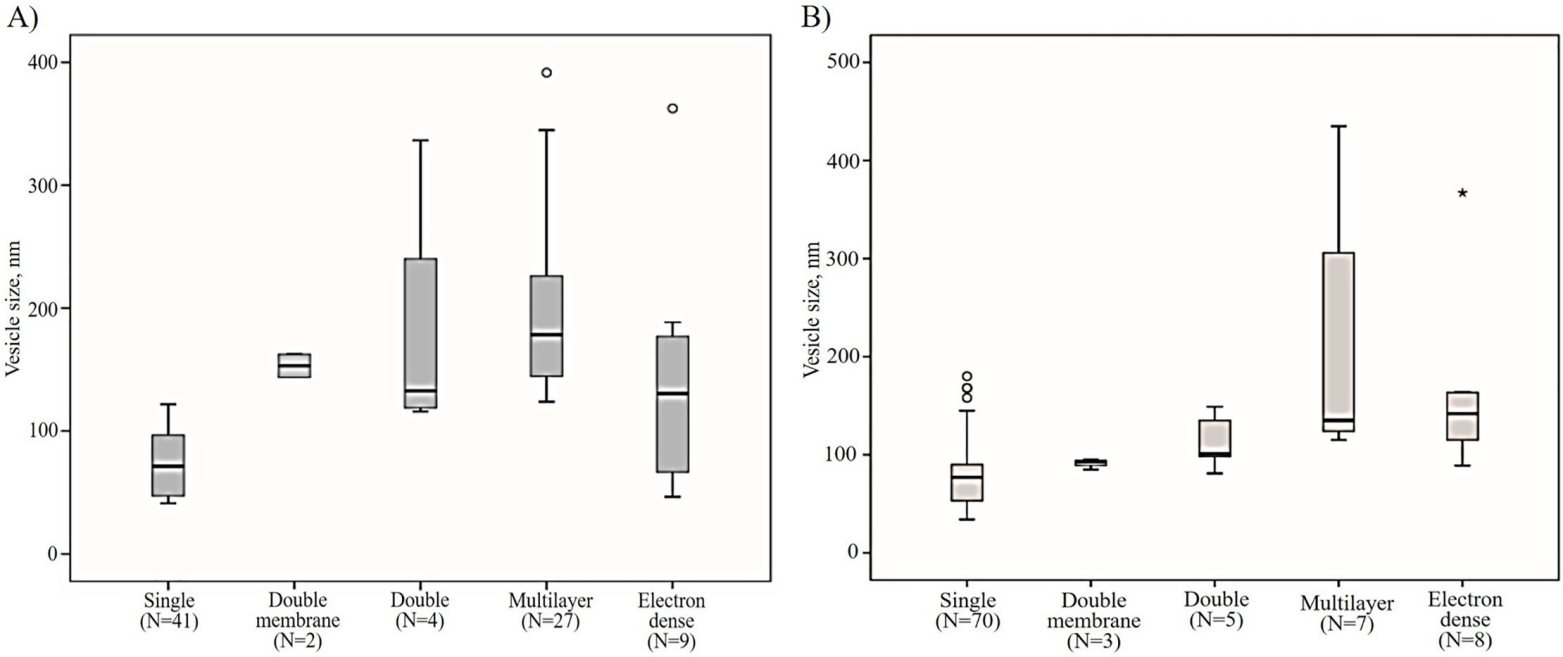
Representation in size of EVs with various morphology. Single, double, double membrane, multilayer and vesicles with electron dense cargo were visualized by cryo-EM both in vesicle samples of pooled CSF from (A) PD patients and (B) individuals of comparison group. N is the number of particles of the indicated morphological type.

Morphological characteristics of EVs were performed via cryo-EM in CSF from PD patients and from individuals of the comparison group. Cryo-electron microscopy allowed us to visualize EVs of various sizes and morphology with lipid bilayers and vesicle internal structures. In total images of 84 particles were analyzed for CSF samples from PD patients and 102 particles for the samples from individuals of the comparison group. Most of the visualized particles could be classified as single, double, double membrane, multilayer and vesicles with electron dense cargo. The particles varied in size from 26 to 392 nm and from 34 to 435nm in CSF samples of PD patients (Fig 2A) and individuals of comparison group, respectively (Fig 2B).

**Fig 3:**
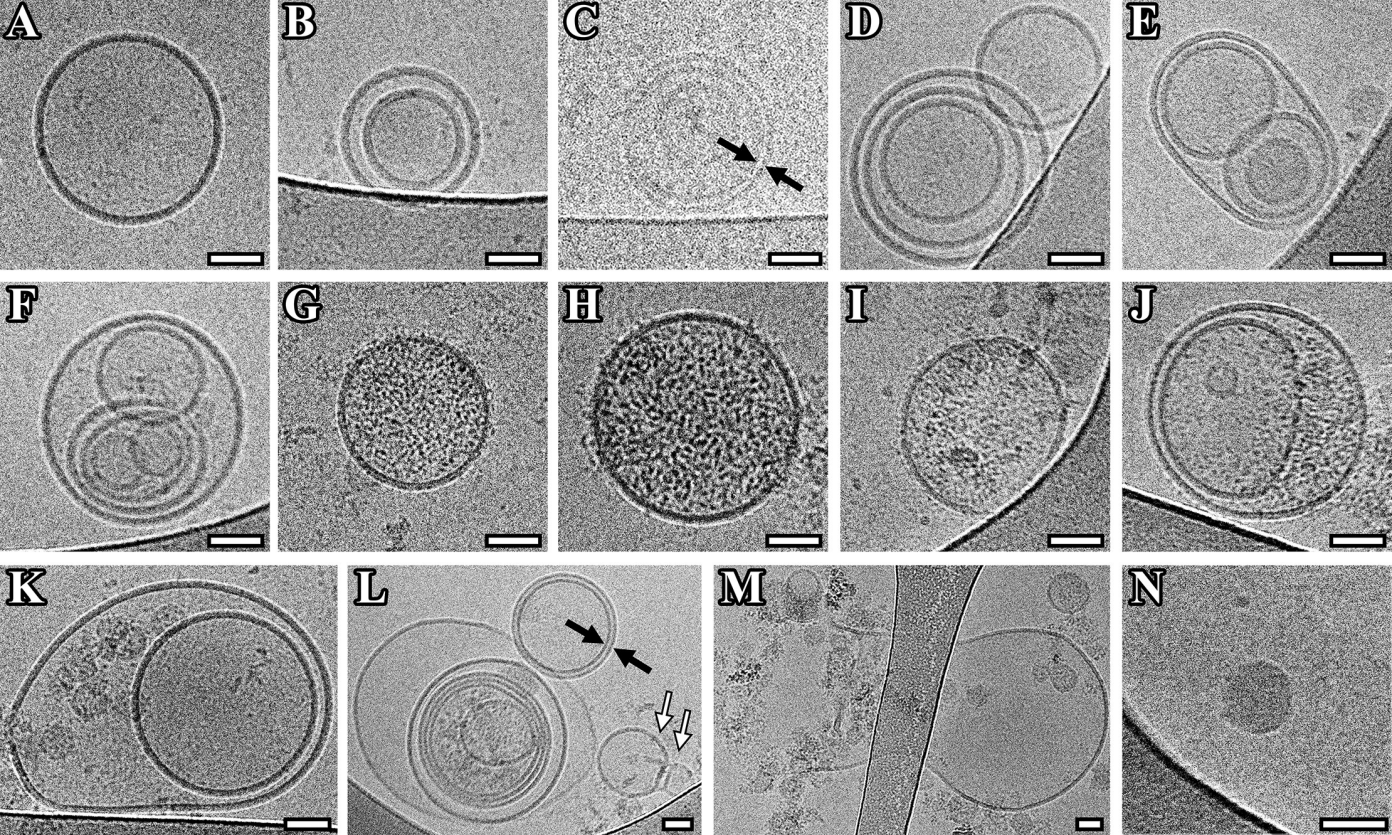
Cryo-EM images of EVs isolated from pooled CSF of Parkinson's disease patients. (A) Single vesicles; (B, K) double vesicles; (C, L) double-membrane vesicles; (C-F, L) multilayer vesicles; (G-J, L) vesicles with electron dense cargo in lumen; (K, M) vesicles with broken membrane. The arrows point to vesicle with double membrane (black arrow) and “bowling pin” vesicle (white arrow). Scale bars are 50 nm.

More than 80 percent of the particles were classified as vesicles due to the clear presence of lipid bilayer/membrane. Most of the vesicles were intact and had a round shape. Single (75±27 nm) (Fig 3A, 3G, 3H and 3J), double (179±105 nm) (Fig 3B) and multilayer vesicles (206±78) (Fig 3C–3F) were visualized in the sample of isolated vesicles from pooled CFS of PD patients. Vesicles were assigned into multilayer category when two or more vesicles were contained inside a larger one. Various combinations, having from two to six smaller vesicles inside the larger one, were found (Fig 3C–3F, 3I and 3L).

All multilayer vesicles were delimited by a membrane bilayer and had either round or slightly elongated shape. Among observed variants the vesicles containing two smaller vesicles in one of which there is a smaller vesicle were visualized (Fig 3E). Another variant was in one vesicle there are two others, in one of which there is a smaller vesicle, having two smaller vesicles both inside the same large one (Fig 3F). Interestingly, we found that the size of multilayer vesicles (179 (124–392) nm) was larger than one of the single vesicles (71 (41–122) nm) (p < 0.0001) (Fig 2A).

Some vesicles showed a damaged membrane and were surrounded by electron-dense material (Fig 3K). In one case a large vesicle with broken membrane and smaller intact vesicles released from it was observed (EV size was about 1 μm) (Fig 3M). In addition, vesicles filled with electron-dense material in their lumen were found (140±97 nm) (Fig 3G–3J and 3L).

In CSF sample, besides double vesicles, vesicles with two membranes bilayers were found, which fit tightly to each other around the whole circumference (153±13 nm) (Fig 3C and 3L). The average width of the double membrane was 11.7±0.1 nm.

Also, we found double vesicles, in which the outer vesicle contains electron dense material (cargo) and carries inside its lumen cytoplasm another smaller electron translucent vesicle deformed into half-moon shape (Fig 3I). Other multilayer vesicles in which the penultimate and the last in size vesicles contain electron dense material were revealed. In that case, the smallest vesicle was deformed into a bean (Fig 3L). Unusually shaped vesicle in the form of bowling pin was also observed (Fig 3L).

A part of visualized particles was round, with high electron dense and without a visible lipid bilayer (Fig 3K and 3N). They had a wide range in size distribution (from 25 to 41 with average size 35±5 nm). Based on these characteristics, it can be assumed that these particles are lipoproteins. Protein aggregates outside EVs were also visualized.

**Fig 4:**
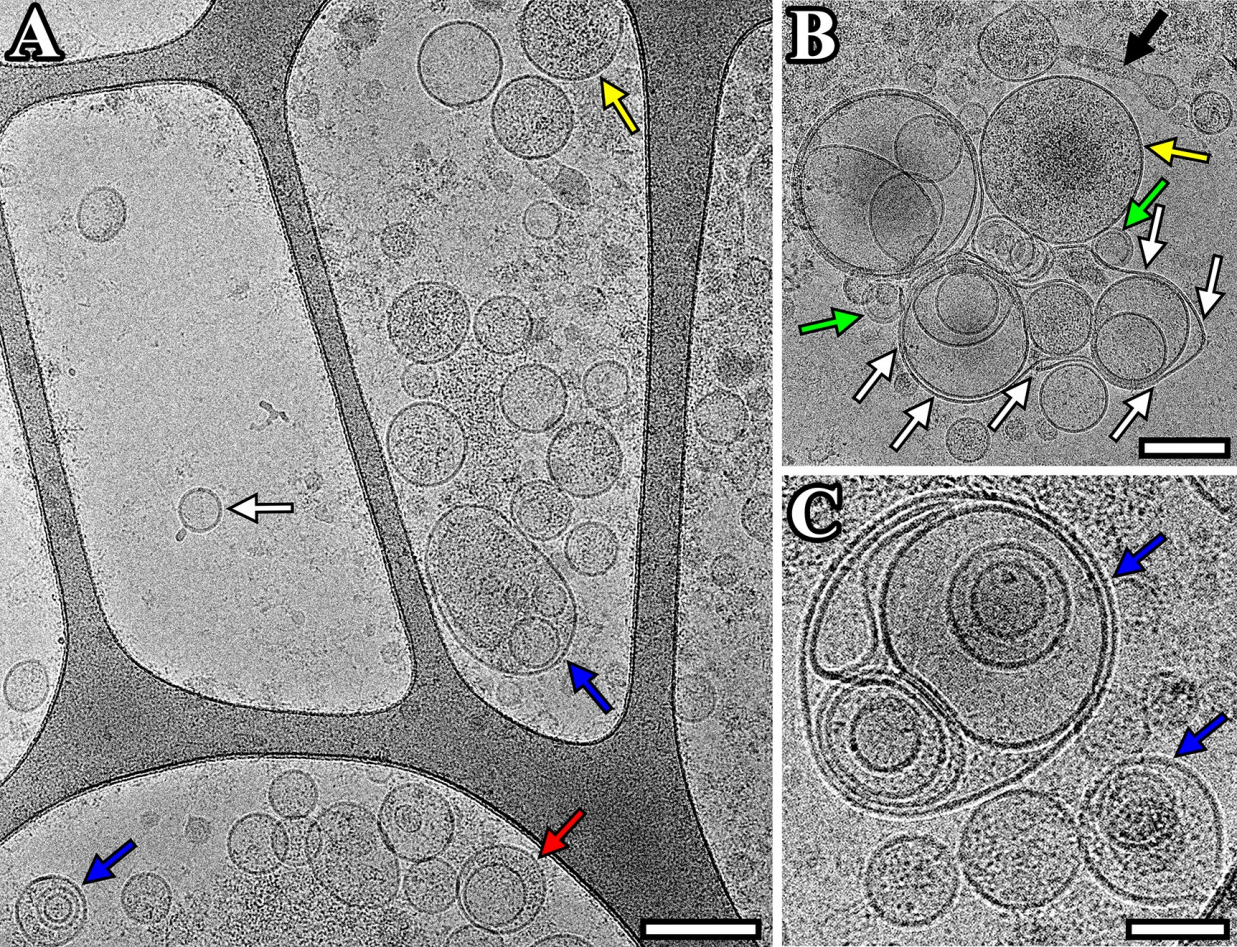
Cryo-EM images of EVs isolated from pooled CSF sample from individuals of the comparison group. (A) Micrograph of single (white arrow), double (red arrow), multilayer (blue) vesicles and vesicles with electron dense cargo in lumen (yellow arrow); (B) micrograph of double-membrane vesicles (green arrow), vesicles with electron dense cargo in lumen (yellow arrow), vesicle in form of sack (white arrows), distinctly elongated vesicle (black arrow); (C) micrograph of multilayer vesicles (blue arrows). Scale bars are 200 nm for figures A, B and 100 nm for figure C.

In CSF sample from individuals of the comparison group we visualized the single (81±35 nm), double (113±28 nm) and multilayer (221±129 nm) vesicles, vesicles filled with electron-dense material (162±88 nm) and lipoprotein particles (31±10 nm) (Fig 4A–4C). The size of multilayer vesicles (135 (115–435) nm) was larger than that of the single vesicles (77 (34–180) nm) (p < 0.0001) (Fig 2B). In addition, we found a multilayer vesicle that contains another non-round shaped vesicle in the form of virgule (Fig 4C). A large vesicle (687 nm) was revealed with a deformed outer membrane in the form of sack, that contains several smaller size vesicles inside (Fig 4B). Also, a distinctly elongated vesicle was visualized (Fig 4B). Similar to pooled CSF sample of PD patients, this CSF sample contained vesicles with two membrane bilayers (91±5 nm). The average width of the double membrane bilayer in such vesicles was 11.9±1.0 nm.

## Discussion

In this study, we evaluated the morphology, size, phenotype and concentration of EVs isolated from CSF. Up to our knowledge, only one study has so far examined EVs from CSF by cryo-electron microscopy [23] and none in PD patients. It’s worth noting, that CSF interacts directly with the extracellular space and neighboring microenvironment within the brain and potentially can reflect changes in both normal physiology and in disease pathology. Therefore, EVs from CSF are now considered as an ideal biomarker source for brain disorders. In addition, there is the evidence that CSF vesicles may mediate neurological diseases.

In our study NTA technique based on the Brownian motion of particles allowed us to determine the size distribution and concentration of EVs isolated from CSF. The size of the observed vesicles and the presence of protein exosomal marker on the membrane confirmed by flow cytometry, suggests that most of the vesicles in the sample were represented as exosomes. The detected size of the vesicles and their concentration evaluated using NTA was in accordance with previous data [7, 23]. It should be noted that the obtained concentration of CSF vesicles is two orders of magnitude lower than that described for peripheral blood plasma [22, 24].

EVs of various sizes and morphology with lipid bilayers and vesicle internal structures were observed by cryo-electron microscopy. We showed that EVs from CSF have a round shape, which is consistent with the data obtained for other human biofluids and cell culture conditioned medium using cryo-EM [6, 13–17, 21]. Previous studies utilizing the conventional transmission electron microscopy suggested that vesicles have a cup-shaped morphology [25]. Sample preparation technique has been found to affect the shape of EVs. It was shown that the sample dehydration, addition of heavy metals as contrasting agents and drying before imaging may lead to vesicle collapse, resulting in its cup-shaped morphology [25]. Oppositely, in cryo-EM the sample is quickly vitrified thus preventing water crystal formation and remains fully hydrated while imaging, which allows to perform the sample visualization in nearly native state [1, 12, 26–28]. However, the specimen preparation methods (blotting prior to vitrification) for cryo-EM could influence on the number and size distribution of EVs as large or small particle could be selectively enriched or extracted during sample preparation.

Single, double, double with two membrane bilayers and multilayer vesicles were revealed in the present study. Such a variety of vesicles was in accordance with previously obtained data for other human biological fluids and cell culture conditioned medium using cryo-EM [6, 13–17]. We found that multilayer vesicles were larger than the single vesicle (p<0.0001). The same results were received earlier for EVs purified from conditioned media of cultured human cells by cryo-EM technique [17]. We also visualized double and multilayered vesicles containing electron dense material, while most vesicles were electron transparent. EVs that called dark vesicles were described earlier in ejaculate [15]. However, in contrast to this study, here the dark substance surrounding one secondary vesicle did not deform the membranes of the outer vesicle, which may indicate an increased dark substance density in this vesicle. In addition, EVs with compromised membrane integrity were identified. That could be explained by possible breakage occurred during sample preparation.

In our study a multilayer vesicle with deformed vesicle inside and the vesicle in form of sack were revealed in the CSF sample from individuals of comparisons group. Similar vesicles have been shown in human ejaculate samples [16].

Previously, it was suggested that the visualized multilayered structures could be artifacts caused by the centrifugation [29]. Several studies have reported that centrifugation may cause aggregation or morphological changes of EVs [18, 29, 30], which could lead to erroneous conclusions about vesicle composition or phenotype [31]. However, observed structures are unlikely to be the result of an artifact caused by purification procedures, since in other study very similar morphology of the vesicles was also described for unprocessed EVs from ejaculate [16] and when another procedure for isolation of EVs was used [32].

It is also noteworthy that the average size of EVs according to NTA and cryo-EM analysis was similar. However, large vesicles sized from 0.3 to 1 μm were revealed via cryo-EM. This could be explained by the fact that in NTA the settings optimal for detecting exosome range size (40–150 nm) [2] are not compatible for tracking larger vesicles or particles. On the other hand, the scattering intensity of these large structures was so intense that they were considered as noise and excluded from the analysis [31]. We found that ultracentrifugation pellets contain not only EVs but other contaminants like protein aggregates and lipoproteins of density similar to that of EVs, that could be result of ultracentrifugation [33–35].

Protein aggregates were shown to affect the morphology of vesicles [36]. In experiments on cells, it was found that prions could increase the number of triple-membrane compare to single-membrane exosomes. It is worth noting that more data appears that alpha-synuclein has prion transmission properties [2] leading to the spread of alpha-synuclein pathology. Moreover, the presence of alpha-synuclein in EVs has previously been shown both for cell cultures and for EVs derived from biofluids [2, 7]. Although we did not find protein filamentous in EVs similar to those found by Davide Zabeo and co-authors [17] there is a need to study the relationship between alpha-synuclein transmission from cell to cell and morphology/ size of EVs.

## Conclusion

Thus, in our study, we described the characteristics of CSF vesicles and found their variety suggesting that subpopulations of EVs with different and specific functions may exist. Our findings show a necessity for a new way of defining subpopulations of EVs that could help to improve the interpretation of experimental results and widen our general understanding of the biological functions of EVs, especially exosomes.
